# Ventricular Septal Defect from Takotsubo Syndrome

**DOI:** 10.1155/2016/2693062

**Published:** 2016-08-03

**Authors:** Daniel Y. Lu, Julie Caplow, Neha Quatromoni, Rhondalyn Forde-McLean, Anjali Tiku Owens

**Affiliations:** ^1^Department of Internal Medicine, Hospital of the University of Pennsylvania, Philadelphia, PA 19104, USA; ^2^Division of Cardiology, Hospital of the University of Pennsylvania, Philadelphia, PA 19104, USA

## Abstract

Takotsubo Syndrome is a transient condition characterized by left ventricular systolic dysfunction with apical akinesis/dyskinesis and ballooning. Although the prognosis with medical management is excellent in most cases, rare cases of serious complications can occur. We present here a case of a 71-year-old woman presenting with acute decompensated heart failure with initial findings consistent with a myocardial infarction, who was found instead to have an acute ventricular septal defect as a complication of Takotsubo Syndrome.

## 1. Introduction

Takotsubo Syndrome [1], also known as Takotsubo cardiomyopathy, apical ballooning syndrome, or stress-induced cardiomyopathy, is an increasingly recognized syndrome characterized by transient systolic dysfunction and EKG/cardiac enzyme changes that can mimic myocardial infarction. Echocardiography often reveals a characteristic pattern including apical akinesia/dyskinesia and ballooning along with basal hyperkinesia [2–5]. Although this condition is usually associated with an excellent prognosis and recovery with medical management in most patients [5, 6], serious complications can occur, including most frequently pulmonary edema and cardiogenic shock and less frequently cardiac rupture. We present here a case of a patient with an acute ventricular septal defect as a complication of Takotsubo Syndrome.

## 2. Case Presentation

**Figure 1:**
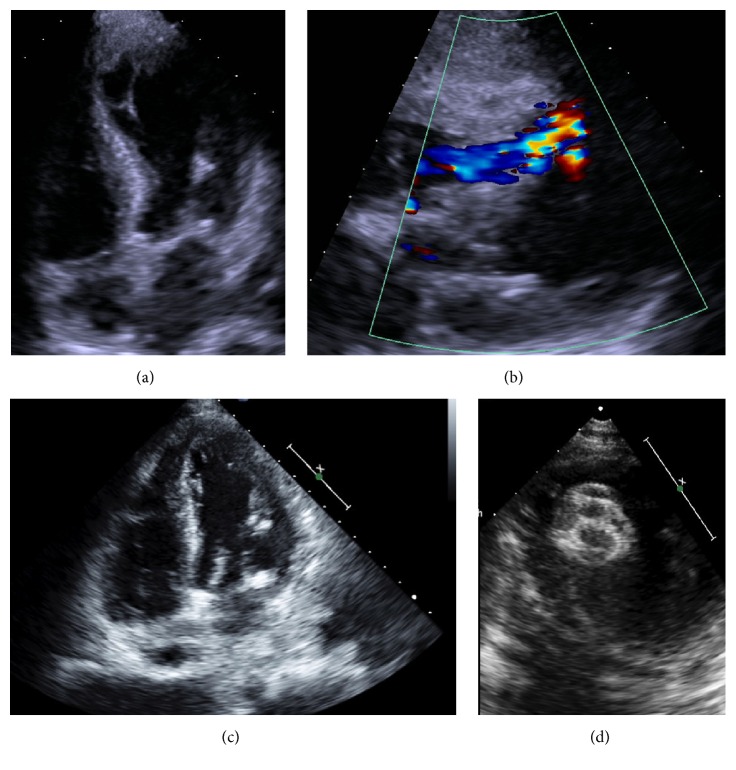
(a) Outside hospital TTE. Apical 4 chamber view demonstrating left ventricular apical ballooning. (b) Outside hospital TTE. Subcostal view demonstrating VSD with left to right shunt. (c) TTE 11 days later with recovery of left ventricular morphology. (d) Amplatzer in place after repair of VSD.

The patient was a 71-year-old woman with diabetes, hypertension, and cirrhosis, transferred to our hospital for management of acute decompensated heart failure. She was previously a highly active and independent individual until a recent tibial/fibular fracture from a mechanical fall. Her home medications were Insulin, Tramadol, and Bactrim. She presented from her rehab facility to an outside hospital with hyperglycemia (glucose greater than 700) and, on initial evaluation, was noted to have ST elevations in V3–V6, Troponin 4.46, and creatine kinase 130. She was diagnosed with a subacute myocardial infarction (MI). Transthoracic echocardiogram (TTE) revealed a left ventricular ejection fraction (EF) of 20–25%, with apical akinesia and ballooning and hyperdynamic base (Figure 1(a), Video 1 in Supplementary Material available online at http://dx.doi.org/10.1155/2016/2693062) consistent with Takotsubo Syndrome, as well as a distal muscular ventricular septal defect (VSD) (Figure 1(b), Video 2). She received intravenous (IV) diuresis, was initiated on IV heparin and a heart failure regimen, and was transferred to our hospital for further management.

On arrival, she was afebrile with blood pressure of 107/51 mmHg, heart rate of 97 beats per minute, and respiratory rate of 18 on 3 L of oxygen. On examination she had a 3/6 holosystolic murmur loudest at the apex and was volume overloaded with jugular venous distension to 15 cm H_2_O and 2+ lower extremity edema. EKG showed normal sinus rhythm, poor R-wave progression, T-wave inversions in II, III, aVF, V2–V6, and prolonged QTc of 570 ms, patterns consistent with those described with Takotsubo Syndrome [2–4, 7, 8]. Repeat TTE on arrival, 11 days after the initial TTE, showed EF recovery to 75% (Figure 1(c), Video 3), a persistent 1 cm inferior apical VSD, and right ventricular (RV) dilatation and elevated pulmonary pressures, with pulmonary artery systolic pressure (PASP) of 57 mmHg. The VSD was thought to be a major contributor to her continued decompensation. She was managed with diuresis and medical optimization and underwent evaluation for VSD repair.

Although she had no prior echocardiogram for comparison, discussion with the patient's primary care provider revealed that she had no cardiac history and no documented cardiac murmur throughout her prior history. To rule out VSD as a complication of MI, the patient underwent left heart catheterization, demonstrating completely normal coronary arteries. Ventriculography confirmed VSD. Right heart catheterization demonstrated right atrial pressure of 7 mmHg, pulmonary artery pressure of 42/14 mmHg, wedge pressure of 10 mmHg, cardiac output of 4.0 L/min, and index of 2.7 L/min/m^2^, with Qp : Qs ratio of 2.2 : 1, consistent with a large VSD. Given these findings, initial echocardiographic appearance of her heart, and rapid EF recovery, she was thought to have a rare case of ventricular septal perforation due to Takotsubo Syndrome. She underwent transcatheter closure of her VSD (Figure 1(d), Video 4), was medically optimized afterwards, and was eventually discharged home after a prolonged hospitalization. Repeat echocardiography performed 2 months later demonstrated normal PASP and improvement in right heart function.

## 3. Discussion

Takotsubo Syndrome is a condition of transient left ventricular failure that can mimic acute MI. It predominantly affects older women and is often associated with physical or emotional stressors [3, 5–8]. Although the exact pathogenesis is unknown, the role of catecholamine surges coupled with reduced parasympathetic modification resulting in myocardial stunning, multivessel epicardial spasm, and microvascular spasm has been implicated [2, 3, 8–10]. While Takotsubo Syndrome has an excellent prognosis with mostly complete recovery of cardiac function, it can occasionally lead to severe complications such as cardiac rupture, including left ventricular free wall rupture [3, 11]. While the mechanism is unclear, reports of cardiac rupture from Takotsubo Syndrome have included discovery of inflammatory infiltrates as well as myocyte and contraction-band necrosis at the rupture site, which may potentially be in part secondary to the aforementioned high catecholamine state [5, 11–13].

As demonstrated in this case, echocardiography can be critical in the diagnosis and followup of Takotsubo Syndrome, often being the first imaging modality to identify the pattern of wall motion classically associated with the syndrome as well as other atypical variants, and is important for followup to recovery. Echocardiography is also additionally useful both for evaluating for possible complications that could significantly alter management (i.e., thrombus, cardiac rupture, mitral regurgitation, and outflow tract obstruction) and to identify imaging characteristics (i.e., EF, E/e' ratio, and mitral regurgitation) that may prognosticate poor outcomes [10, 14]. Other more advanced echocardiography techniques that can be used include speckle-tracking, contrast echo, and stress echo, which can provide further understanding regarding overall pathophysiology and mechanisms of dysfunction in this syndrome [10].

Our patient, previously healthy, presented with acute decompensated heart failure, demonstrating the classic features of Takotsubo Syndrome: demographic of a postmenopausal woman with recent stressor of hospitalization, surgery, and rehabilitation for fractures, TTE findings of akinesis and ballooning of the left ventricular apex with basal hyperkinesis, and EKG and Troponin abnormalities mimicking an ST-elevation MI in the absence of angiographic coronary artery disease. Although she was initially diagnosed with a subacute MI at the outside hospital, her case was not consistent with infarction for several reasons: most importantly, her coronaries were completely normal without any stenosis or luminal irregularities, there were no Q-waves present on EKG on followup, and postrecovery echocardiograms demonstrated normal wall thickness without evidence of residual wall motion abnormalities. Although she had no prior TTEs for comparison, her VSD was not consistent with a congenital or chronic VSD for several reasons: she was 71 years old, had no prior cardiac history, and was functionally normal prior to presentation, an unlikely scenario for a VSD of this size. Furthermore, she presented with a presumably new loud systolic murmur, given her previously documented normal cardiac exams. Finally, a large chronic VSD in a 71-year-old adult would have likely led to some degree of chronic pulmonary vascular remodeling. Although this patient presented with elevated pulmonary pressures, this resolved to normal soon after VSD closure. Given these reasons in addition to her angiographically normal coronaries, the etiology of her acute VSD is likely secondary to ventricular septal perforation due to Takotsubo Syndrome.

Acute ventricular septal defect is a known serious complication of acute MI and can result in significant mortality rates of over 90% if managed medically [15, 16]. The mechanism of rupture in acute MI is thought to be related to transmural necrosis and possibly myocardial hemorrhage. Furthermore, VSD in acute MI is fundamentally different from a congenital VSD given its acuity and its mechanism involving tissue necrosis that causes and surrounds the defect, which may make intervention technically challenging [15]. Given the acuity and severity of disease and high risk of morbidity, surgical repair or, in recent years, percutaneous repair is crucial [16, 17]. Although VSD development after Takotsubo Syndrome is incredibly rare and not as well understood, its acuity as well as its pathology of necrosis and inflammation in select reports [5, 11] likely portends a similarly poor prognosis without intervention.

Overall, our case highlights the importance of recognizing, associating, and treating VSD as a complication of Takotsubo Syndrome, despite the common understanding that it carries a benign prognosis, and demonstrates that complications such as this can be significant clinically even with resolution of the Takotsubo Syndrome itself.

## Supplementary Material

Supplemental Video 1 shows the initial outside hospital echocardiogram's apical 4-chamber view demonstrating apical akinesis and ballooning with hyperdynamic base, features consistent with Takotsubo Syndrome. Supplemental Video 2 shows the initial outside hospital echocardiogram's subcostal view demonstrating VSD with left to right shunt. Supplemental Video 3 shows our echocardiogram, 11 days later, with recovery of left ventricular morphology and ejection fraction. Supplemental Video 4 shows the echocardiogram after repair of VSD with Amplatzer in place.

## References

[1] Lyon A. R., Bossone E., Schneider B. (2016). Current state of knowledge on Takotsubo syndrome: a Position Statement from the Taskforce on Takotsubo Syndrome of the Heart Failure Association of the European Society of Cardiology. *European Journal of Heart Failure*.

[2] Prasad A., Lerman A., Rihal C. S. (2008). Apical ballooning syndrome (Tako-Tsubo or stress cardiomyopathy): a mimic of acute myocardial infarction. *American Heart Journal*.

[3] Templin C., Ghadri J. R., Diekmann J. (2015). Clinical features and outcomes of takotsubo (stress) cardiomyopathy. *The New England Journal of Medicine*.

[4] Yoshikawa T. (2015). Takotsubo cardiomyopathy, a new concept of cardiomyopathy: clinical features and pathophysiology. *International Journal of Cardiology*.

[5] Bybee K. A., Prasad A. (2008). Stress-related cardiomyopathy syndromes. *Circulation*.

[6] Kono T., Sabbah H. N. (2014). Takotsubo cardiomyopathy. *Heart Failure Reviews*.

[7] Gianni M., Dentali F., Grandi A. M., Sumner G., Hiralal R., Lonn E. (2006). Apical ballooning syndrome or takotsubo cardiomyopathy: a systematic review. *European Heart Journal*.

[8] Wittstein I. S., Thiemann D. R., Lima J. A. C. (2005). Neurohumoral features of myocardial stunning due to sudden emotional stress. *The New England Journal of Medicine*.

[9] Norcliffe-Kaufmann L., Kaufmann H., Martinez J., Katz S. D., Tully L., Reynolds H. R. (2016). Autonomic findings in takotsubo cardiomyopathy. *The American Journal of Cardiology*.

[10] Citro R., Lyon A. R., Meimoun P. (2015). Standard and advanced echocardiography in takotsubo (stress) cardiomyopathy: clinical and prognostic implications. *Journal of the American Society of Echocardiography*.

[11] Kumar S., Kaushik S., Nautiyal A. (2011). Cardiac rupture in takotsubo cardiomyopathy: a systematic review. *Clinical Cardiology*.

[12] Ohara Y., Hiasa Y., Hosokawa S. (2005). Left ventricular free wall rupture in transient left ventricular apical ballooning. *Circulation Journal*.

[13] Sacha J., Masełko J., Wester A., Szudrowicz Z., Pluta W. (2007). Left ventricular apical rupture caused by takotsubo cardiomyopathy—comprehensive pathological heart investigation. *Circulation Journal*.

[14] Citro R., Rigo F., D'Andrea A. (2014). Echocardiographic correlates of acute heart failure, cardiogenic shock, and in-hospital mortality in tako-tsubo cardiomyopathy. *JACC: Cardiovascular Imaging*.

[15] Crenshaw B. S., Granger C. B., Birnbaum Y. (2000). Risk factors, angiographic patterns, and outcomes in patients with ventricular septal defect complicating acute myocardial infarction. GUSTO-I (Global Utilization of Streptokinase and TPA for Occluded Coronary Arteries) Trial Investigators. *Circulation*.

[16] Risseeuw F., Diebels I., Vandendriessche T., De Wolf D., Rodrigus I. E. (2014). Percutaneous occlusion of post-myocardial infarction ventricular septum rupture. *Netherlands Heart Journal*.

[17] Assenza G. E., McElhinney D. B., Valente A. M. (2013). Transcatheter closure of post-myocardial infarction ventricular septal rupture. *Circulation: Cardiovascular Interventions*.

